# Serum Uric Acid Level as an Estimated Parameter That Predicts All-Cause Mortality in Patients with Hemodialysis

**DOI:** 10.3390/jpm15070305

**Published:** 2025-07-11

**Authors:** Sheng-Wen Niu, I-Ching Kuo, Yen-Yi Zhen, Eddy Essen Chang, Li-Yun Chang, Chung-Ting Cheng, Hugo You-Hsien Lin, Yi-Wen Chiu, Jer-Ming Chang, Shang-Jyh Hwang, Chi-Chih Hung

**Affiliations:** 1 Graduate Institute of Clinical Medicine, College of Medicine, Kaohsiung Medical University, Kaohsiung 80708, Taiwan; 950138kmuh@gmail.com (S.-W.N.); 980135kmuh@gmail.com (I.-C.K.); sjhwang@kmu.edu.tw (S.-J.H.); 2 Division of Nephrology, Department of Internal Medicine, Kaohsiung Medical University Hospital, Kaohsiung Medical University, Kaohsiung 80708, Taiwan; a0928306234@gmail.com (Y.-Y.Z.); eotaxin002@yahoo.com.tw (E.E.C.); yunchang1104@gmail.com (L.-Y.C.); xg3636@gmail.com (C.-T.C.); yukenlin@yahoo.com.tw (H.Y.-H.L.); chiuyiwen@gmail.com (Y.-W.C.); jemich@kmu.edu.tw (J.-M.C.); 3 Division of Nephrology, Department of Internal Medicine, Kaohsiung Medical University Gangshan Hospital, Kaohsiung 820111, Taiwan; 4 Department of Medicine, College of Medicine, Kaohsiung Medical University, Kaohsiung 80708, Taiwan; 5 Department of Medical Research, Kaohsiung Medical University Hospital, Kaohsiung Medical University, Kaohsiung 80708, Taiwan; 6 Regenerative Medicine and Cell Therapy Research Center, Kaohsiung Medical University, Kaohsiung 80708, Taiwan

**Keywords:** uric acid, all-cause mortality, hemodialysis, Charlson comorbidity index

## Abstract

**Background**: Serum uric acid (UA) in end-stage kidney disease (ESKD) patients serves as a critical indicator for nutrition and inflammation, showing a U-shaped association with all-cause mortality. **Methods**: Our study assessed UA’s survival predictive value in 2615 ESKD patients, stratified by the Charlson Comorbidity Index (CCI) into groups of <4 (*n* = 1107) and ≥4 (*n* = 1508). **Results**: Cox regression revealed distinct patterns. For ESKD patients with CCI < 4, UA levels > 8.6 mg/dL were a mortality risk factor (HR: 1.61, 95% CI: 1.01–2.38) compared to 7.1–7.7 mg/dL. Conversely, in patients with CCI ≥ 4, UA levels < 5.8 mg/dL were a mortality risk factor (HR: 1.53, 95% CI: 1.20–1.95) compared to >8.6 mg/dL. **Conclusions**: Higher serum UA in ESKD patients with high comorbidities (CCI ≥ 4) is not a risk factor. Low UA should be prevented across all ESKD patients. A personalized approach using CCI and corresponding serum UA levels offers a key reference for managing UA in hemodialysis patients.

## 1. Introduction

Protein–energy wasting (PEW), which is a pathological entity that is characterized by low protein intake and low deposits of energetic materials, contributes to high mortality in patients with chronic kidney disease (CKD) who are undergoing maintenance hemodialysis (HD) [1]. Malnutrition and low serum albumin are risk factors for all-cause mortality in patients with CKD [2] and ESKD, which is frequently associated with systemic inflammation. Subsequently, the uremic milieu elicits oxidative stress and systemic inflammation, both promoting vascular aging via endothelial dysfunction and vascular calcification, contributing to cardiovascular disease and cardiovascular mortality [3].

Higher inflammation and nutritional parameters associated with PEW, such as cholesterol, sugar, and UA, in patients with ESKD are the pathological causes of mortality [4]. The statistical relationship of HbA1c levels in serum and all-cause mortality displays a U-shaped curve in HD patients, especially identified in cardiovascular mortality [5]. Hsuan Chiu et al. also reported a U-shaped association between low-density lipoprotein cholesterol (LDL) and all-cause and cardiovascular mortality in patients with stage 3–5 CKD [6]. As mentioned above, serum UA levels are reflected in both inflammation, nutritional parameters and anti-oxidant response [4]. It has been documented in the literature that a U-shaped relationship exists between serum UA levels and all-cause mortality in patients receiving maintenance dialysis [7].

Hyperuricemia or gouty attacks in dialysis patients could also be a reflection of the underlying inflammatory state, with subsequent increased risk of all-cause and cardiovascular mortality. UA, however, is also a nutritional factor [7] and, along with hypouricemia, is linked to malnutrition–inflammation–atherosclerosis (MIA) syndrome [8]. Contrary to the general population, low serum UA is associated with higher all-cause mortality in dialysis patients, especially in those with PEW [9]. Patients with hyperuricemia possess higher antioxidant capacity and, therefore, less oxidative damage and better nutritional status [4]. A previous study evidenced that hyperuricemia can merely predict renal outcome in patients with CKD without metabolic syndrome (MS) or diabetes mellitus (DM) [10]. Also, mounting evidence has noted that urate-lowering therapy is likely to improve the incidence of stroke without comorbidities [11]. As mentioned above, elevated UA in serum signifies the elevation of inflammation in patients with fewer comorbidities and exacerbates overall mortality, but in patients with more comorbidities, differences in recovery or nutritional condition may be exhibited and may relate to lower overall mortality. Several studies have investigated the relationship between comorbidity and mortality in patients with CKD during dialysis, and they widely used the CCI (Table S1) to predict mortality [12]. Based on the criteria, the subjects of this study were mainly categorized as having fewer or more comorbidities according to the CCI, and we aimed to investigate the relationship between serum UA levels and total mortality in the two individual groups.

While gout treatment typically involves medication based on disease severity, for dialysis patients with multiple comorbidities, elevated serum UA may serve as a nutritional index rather than an inflammatory one. This suggests that specific treatment might not always be required. However, to mitigate risks, urate-lowering agents can be beneficial. We hypothesized that a personalized approach using CCI and corresponding serum UA levels offers a key reference for managing UA in hemodialysis patients. Therefore, we questioned whether hyperuricemia is a risk factor associated with worse survival in dialysis patients and whether comorbidities, surveyed with the CCI, could modify the effect of hyperuricemia.

## 2. Materials and Methods

### 2.1. Study Design and Participants

This study (the Kaohsiung Hemodialysis Study) was designed as a mixed study with retrospective (2002–2009, and then followed until 23 November 2014) and prospective (from 23 September 2014 to 31 December 2014) cohorts to assess the quality of patient care on the basis of the Hemodialysis Operation Plan Executive System developed by the Taiwan Society of Nephrology. In total, 2848 consecutive incident hemodialysis patients who underwent dialysis thrice a week were recruited from three affiliated hospitals and nine associated hemodialysis clinics of Kaohsiung Medical University, Southern Taiwan, between 11 November 2002 and 31 May 2009, and followed until 31 December 2014. Of these, 95 patients underwent hemodialysis for less than 90 days (31 withdrew, 20 died, and 44 had paused hemodialysis), 5 were aged less than 18 years, 94 patients were lost to follow-up in less than 6 months, and more than 10% of the data were missing for 39 patients. Therefore, the final study population consisted of 2615 incident hemodialysis patients.

### 2.2. Collection of Demographic, Medical, and Laboratory Data

Dialysis initiation was performed according to the regulations of the NHI Administration of Taiwan, which stipulated the required laboratory data, nutritional status, uremic status, and eGFR. The mean eGFR at the start of dialysis was 4.9 mL/min/1.73 m^2^, and the mean residual daily urine volume was 560 mL. Fasting blood samples were analyzed using a COBAS c501 autoanalyzer (Roche Diagnostics, Mannheim, Germany). A total of 178 subjects (6.1%) received peritoneal dialysis during the same period. The NHI administration provides full coverage of HD therapy and erythropoiesis-stimulating agent therapy at a fixed fee. Attending physicians were rotated between dialysis centers, and dialysis machines were involved; artificial kidney and water management applications followed similar procedures.

Furthermore, we did not reduce the surface area of the dialyzer if the Kidney Disease: Improving Global Outcomes (KDIGO) recommended target URR (70%) or Kt/V (>1.4) was achieved. However, if the minimum requirements of URR (>65%) or Kt/V (>1.2) in the Taiwan Nephrology Society guidelines were not met, we increased the surface area of the dialyzer. Ultrafiltration rate was calculated as ultrafiltration volume per body weight. Dialysis efficiency (Kt/V) was assessed monthly using the Daugirdas method (Daugirdas, 1993) [13].

### 2.3. Measurements

Baseline variables included demographic characteristics (age, sex, and year of enrollment), history of DM, congestive heart failure (CHF), hypertension, stroke, cancer, and hepatitis. We obtained and averaged the test results (pre- and post-dialysis BW), laboratory data (serum creatinine, post-dialysis BUN, albumin (methyl bromide) with the Roche cobas^®^ 6000 analyzer (Roche Diagnostics, Mannheim, Germany) and phenol green (bromocresol green (BCG)) albumin determination), white blood cell (WBC) count, heme, total cholesterol, CTR, iron saturation and glucose (AC)), and HD parameters (UF/BW ratio, vascular access, Kt/V (Daugirdas), URR, and nPCR between the 4th and 9th month). Subjects with DM and hypertension were identified through clinical diagnosis. Laboratory data were recorded monthly, and statistical analysis was performed on average data 6 months after stable dialysis. Dialysis adequacy was assessed using the single-cell Daugirdas formula Kt/V = −1n ((postBUN/preBUN) − 0.008 × t) + [(4 − 3.5 × (postBUN/preBUN)) × UF/BW]. The URR was calculated as the ratio of (preBUN − postBUN) as a numerator to the denominator of preBUN (BUN: mg/dL). Post-dialysis BUN values were determined according to the Kidney Disease Outcomes Quality Initiative (KDOQI) guidelines as follows [14]: (1) the ultrafiltration rate was reset to zero, (2) the blood pump was slowed to 100 mL/min for 10–20 s, (3) the pump was stopped, and (4) a sample was withdrawn from the arterial blood line sampling port or from the tubing connected to the arterial needle.

### 2.4. Outcomes

**Figure 1:**
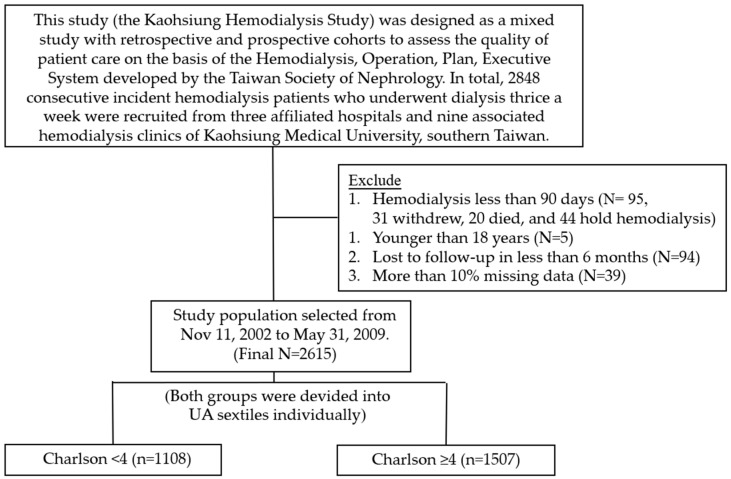
Flow diagram of the study population.

Based on the CCI criteria, all subjects were categorized as CCI < 3 (*n* = 1107) and CCI ≥ 4 (*n* = 1508) (Figure 1), and we divided participants into two groups based on a CCI cutoff of 4 as the median value. The subjects were followed from month 4 of HD to the end of month 20 or death. All-cause mortality was confirmed via a review of death certificates using charts or the National Death Index.

### 2.5. Statistical Analysis

Baseline PA characteristics were assessed as percentages of categorical profiles, alongside the mean ± standard deviation (SD) for continuous variables with approximately normal distribution, and the median and six-quartile range for continuous variables with skewed distribution. The Markov chain Monte Carlo method was applied to minimize the effect of missing covariates (fewer than 5% of values were missing in seven covariates). Multiple linear regression was used to evaluate the relationship between the URR, Kt/V, and the significance factors described in Table 1. The analysis was initially performed without adjustments, which were subsequently applied to several sets of covariates stratified in this study. These models also resolved covariates with *p* < 0.05 in univariate analysis and log-transformed continuous variables with skewed distributions to obtain normal distributions. Age at dialysis initiation, sex, year of entry, DM, hypertension, hepatitis, CHF, post-dialysis BW, nPCR, UF/BW ratio, creatinine, hemoglobin, WBCs, albumin, AC, log-transformed cholesterol, and phosphorus were recorded in this study. Tests were also conducted based on sex, age (≥65 years), DM, CHF, hepatitis, hypertension, anemia (heme < 10 g/dL), albumin (<3.5 g/dL), UF (mean), and BW (mean value). Interactions between subgroups were examined. A *p* value < 0.05 is the threshold for statistical significance. Statistical analyses were performed using R 4.1.3 software (R Foundation for Statistical Computing, Vienna, Austria) and Statistical Product and Service Solutions (SPSS) version 20.0 (SPSS Inc., Chicago, IL, USA).

### 2.6. Ethics Declaration

This study was carried out in accordance with the guidelines of the Declaration of Helsinki. This study was also approved by the Institutional Review Board of Kaohsiung Medical University Hospital (KMUH-IRB-990198).

## 3. Results

### 3.1. Patient Characteristics by UA Quintiles

Table 1 summarizes the baseline clinical and biochemical characteristics of the 2615 participants based on the presence of serum UA levels. The mean age of the participant cohort was 59.1 ± 14.2 years old. Older age; female gender; higher prevalence of DM; lower prevalence of hypertension; a higher CCI; lower levels of albumin, cholesterol, creatinine, potassium, and phosphate; a lower body weight (BW) post-dialysis; a lower UF/BW ratio, blood urea nitrogen (BUN), and pre-dialysis and normalized protein catabolic rate (nPCR); a higher level of ante cibum (AC) glucose, Kt/V (Gotch, K = dialyzer clearance of urea, t = dialysis time, and V = “volume of distribution of urea” approximately equal to “patient’s total body water”); a higher cardiac/thoracic ratio (CTR); and higher all-cause mortality were associated with UA < 5.8 mg/dL. The highest levels of hemoglobin (Hb) were recorded among subjects with UA = 7.1–7.7 mg/dL.

### 3.2. Multivariate Linear Regression for UA

The results of the multivariate linear regression for serum UA (Table 2) indicate that a higher UA level was significantly related to male gender, younger age at dialysis, shorter entry year, lower prevalence of DM, higher pre-dialytic BW, lower Kt/V (Gotch), a higher nPCR, a higher albumin level, a higher log of cholesterol level, a higher phosphate (P) level, and a higher log of the parathydroid (PTH) level. Although some parameters presented different trends of association with UA quintiles, as shown in Table 1, the CCI and serum UA of the subjects were not statistically relevant, as noted in Table 2.

### 3.3. UA Quintiles, Sextiles, and Clinical Outcomes

In the fully adjusted Cox regression (Table S2), the subgroup of UA = 6–7 mg/dL was statistically related to the 39% increase in the risk of all-cause mortality (HR: 1.39, 95% CI: 1.06–1.82) compared with UA > 9 mg/dL with CCI ≥ 4 in the serum UA quintile group. The subgroup of UA > 9 mg/dL was significantly related to an approximately doubled risk of all-cause mortality (HR: 1.99, 95% CI: 1.18–3.36) compared with UA > 9 mg/dL with CCI < 4.

In the fully adjusted Cox regression (Table 3), the subgroup of UA < 5.8 mg/dL was significantly related to a 31% increase in the risk of all-cause mortality (HR: 1.31, 95% CI: 1.06–1.63) compared with UA > 8.6 mg/dL in the UA sextile group. The subgroup of UA < 5.8 mg/dL was significantly related to a 53% increase (HR: 1.53, 95% CI: 1.20–1.95), UA = 6.5–7.1 mg/dL was significantly related to a 37% increase (HR: 1.37, 95% CI: 1.08–1.72), and UA = 7.7–8.6 mg/dL was significantly related to a 34% increase (HR: 1.34, 95% CI: 1.06–1.69) in the risk of all-cause mortality compared with UA > 8.6 mg/dL with CCI ≥ 4. The subgroup of UA > 8.6 mg/dL was significantly related to a 61% increase in the risk of all-cause mortality (HR: 1.61, 95% CI: 1.01–2.38) compared with UA = 7.1–7.7 mg/dL with CCI < 4. As regards these results, we point out the following: (1) The serum UA level could be an indicator of nutritional status in the dialysis subjects, with a high CCI being related to systemic inflammation and energetic exhaustion, so a lower serum UA level is related to higher mortality. (2) In the dialysis subjects with a low CCI, the serum UA level may indirectly reflect inflammation per se. Therefore, a higher serum UA level may be related to higher mortality instead.

Through modeling UA as a continuous variable with restricted cubic splines and plotting it using CCI strata, we found that (1) The lowest HR in all patients was at a UA of 9.3 (Figure S1A). (2) In patients with CCI < 4, the lowest HR was at a UA of 7.9 (Figure S1B). (3) In patients with CCI ≥ 4, the lowest HR was at a UA of 9.4 (Figure S1C).

## 4. Discussion

Hyperuricemia (serum UA levels > 7 mg/dL in men and >6 mg/dL in women) and gout are both common in CKD because of the progressive loss of estimated glomerular filtration rate (eGFR) and renal clearance of UA in patients with CKD or ESKD. In the study of Yang et al., with a larger cohort (*n* = 4242), the prevalence of hyperuricemia was 22.2%, and it was significantly higher in male than in female patients (25.2% vs. 17%, *p* < 0.001) [15]. In the present study, the rate of serum UA level > 7 mg/dL in male patients was 54.7% (719/1315), and the rate of serum UA level > 6 mg/dL in female patients was 48.2% (991/2057) (Table 1). In the fully adjusted model of other variables (Table 2, *n* = 2615), the mean level of UA in female patients was 0.164 mg/dL less than in male (95% CI = −0.283 to −0.045, *p* = 0.007).

Excessive BW (odds ratio (OR) 0.4 [0.2; 0.9]) and Kt/V urea < 1.2 (OR 0.1 [0.04; 0.2]) significantly decrease the efficacy of HD, and they lead to a higher prevalence of hyperuricemia with directionality when only co-occurrence is present [16]. Our study highlights that a higher serum UA level was recorded in patients with lower average age (*p* < 0.001, Table 1). In the fully adjusted model, in patients one year older, the serum UA level was 0.008 mg/dL lower (95% CI = −0.012 to −0.004, *p* < 0.001, Table 2). This result suggests that nutritional status might be the cause. This present study also unveiled that in the fully adjusted model, pre-dialysis BW was 1 kg heavier, the UA level was 0.012 mg/dL higher (95% CI = 0.007 to 0.017, *p* < 0.001); Kt/V was 1.0 lower, and the UA level was 0.524 mg/dL higher (95% CI = −0.804 to −0.243, *p* < 0.001). Furthermore, the serum UA level, albumin level, and nPCR all reflect nutritional status, and a higher UA level, albumin level, and nPCR are related to lower mortality [9]. In the present study, in the fully adjusted model, the nPCR was 1 g/kg/day higher, uric acid was 0.612 mg/dL higher (95% CI = 0.420 to 0.804, *p* < 0.001), the albumin level was 1 mg/dL higher, and the UA level was 0.256 mg/dL higher.

In a retrospective cohort study of 16,057 HD subjects treated at 564 NephroCare centers in EMEA (Europe, Middle East, and Africa; *n* = 15,127) and Latin America (*n* = 930), in terms of laboratory parameters, subjects with higher serum UA levels also had higher values for phosphate, albumin, creatinine, total cholesterol, triglycerides, nPCR, and PTH hormone [7]. Our study has revealed that the UA level is a positive parameter related to the serum phosphate level (Table 1). In the fully adjusted model, the serum phosphate level was 1 mg/dL higher, the serum UA level was 0.267 mg/dL higher, the level of natural log to PTH was 1 higher, the UA level was 0.123 mg/dL higher, the level of natural log to total cholesterol was 1 higher, and the serum UA level was 1.361 mg/dL higher (Table 2). The correlation between the serum UA level and phosphate may be attributed to the efficacy of HD and nutritional status, as with cholesterol.

In patients with ESKD, the total antioxidant capacity was associated with serum UA levels, and lower serum UA levels may result in a reduced total antioxidant capacity in subjects undergoing dialysis [4]. Our study revealed that a higher mortality rate and lower serum UA levels were both correlated overall in subjects with ESKD (compared with UA > 8.6 mg/dL), and the subgroup of UA < 5.8 mg/dL was significantly related to a 31% increase in the risk of all-cause mortality (HR: 1.31, 95% CI: 1.06–1.63) as sextiles (Table 3). In comparison with UA > 9.0 mg/dL, no statistically significant difference exists in the risk of all-cause mortality in other subgroups of different UA levels as quintiles (Table S2). Zawada et al. stated that the relationship of serum UA and all-cause mortality exhibited a U-shaped pattern in a retrospective cohort study of 16,057 HD subjects treated during 2007 to 2016 in NephroCare centers, as documented in the European Clinical Database (EuCliD). This also means that serum UA levels in the ‘low-level’ and ‘high-level’ groups represent a higher risk of mortality than those in the ‘average-level’ group [7]. Similarly, the relationship between dialysis subjects and lipid and cardiovascular mortality has a J-shaped pattern in reverse epidemiology (lower lipid levels and higher vascular events compared to the general population) in 2022 [17]. Herein, we put forward a hypothesis: the relationship between serum UA and mortality at the “high level” and “low level” are distinguished in patients with fewer and more comorbidities. Lower serum UA in patients with more comorbidities and higher serum UA in patients with fewer comorbidities both trend toward higher mortality. We noticed that a different variable, the CCI, led to higher mortality among patients with higher levels and lower levels of serum UA. With respect to the serum UA level and mortality in patients with ESKD, we found that a high UA level was associated with high mortality among patients with CCI < 4, and contrarily, a low UA level was associated with high mortality among patients with CCI ≥ 4.

In the present study, the higher mortality rate associated with higher serum UA levels among patients with CCI < 4 (subgroup of UA > 8.6 mg/dL) was significantly related to a 61% increase in the risk of all-cause mortality (HR: 1.61, 95% CI: 1.01–2.38) compared with UA = 7.1–7.7 mg/dL, shown as sextiles in Table 3, and the subgroup of UA > 9.0 mg/dL was significantly related to an approximately doubled risk of all-cause mortality (HR: 1.99, 95% CI: 1.18–3.36) compared with UA = 7.0–8.0 mg/dL, shown as quintiles in Table S3. It is well known that serum UA levels correlate with many cardiovascular diseases [18] and renal injury without or with few comorbidities [10], including older age, male gender, and hypertension. In our study, we determined significant differences in serum UA levels between male and female patients (*p* < 0.001), as well as between hypertensive and non-hypertensive patients (*p* = 0.0235). There was also a significant negative correlation (*p* < 0.001) between patient age and serum UA levels, suggesting that there are fewer comorbidities in younger patients. Serum UA levels have been considered a risk factor for kidney injury or atherogenic factors that can cause renal inflammation, oxidative stress, endothelial lesions, and hypertension and activate the renin–angiotensin–aldosterone system (RAAS) to provoke cardiovascular disease or renal disease [19]. Elevating the serum UA level promotes the oxygenation of LDL and increases lipid peroxidation and the production of oxygen free radicals (ROS), with subsequent endothelial dysfunction [20] and increased platelet adhesiveness, followed by thrombi formation in HD patients. These processes all evoke the progression of atherosclerosis and increase cardiovascular risk [21]. Although a higher serum UA level was not associated with an increased risk of ischemic heart disease and stroke (Table 1), it was significantly associated with an increased risk of hypertension in this study (*p* = 0.0235, Table 1). Therefore, it is possible that higher levels of serum UA reflect the status of inflammation and may be related to endothelial dysfunction, hypertension, and cardiovascular disease in our HD patients with CCI < 4. Most importantly, however, we should not restrict nutrient supply in those with high UA and high comorbidities.

On the contrary, in patients with CCI ≥ 4, a higher mortality rate was associated with lower serum UA levels. Compared with the subgroup of UA > 8.6 mg/dL, the subgroup of UA < 5.8 mg/dL was significantly related to a 53% increase in the risk of all-cause mortality (HR: 1.53, 95% CI: 1.20–1.95), UA = 6.5–7.1 was significantly related to a 37% increase in the risk of all-cause mortality (HR: 1.37, 95% CI: 1.08–1.72), and UA = 7.7–8.6 was significantly related to a 34% increase in the risk of all-cause mortality (HR: 1.34, 95% CI: 1.06–1.69), presented as sextiles in Table 3. Compared with UA > 9.0 mg/dL, the subgroup of UA = 6.0–7.0 mg/dL was significantly related to a 39% increase in the risk of all-cause mortality (HR: 1.39, 95% CI: 1.06–1.82) as quintiles in Table S2). There are some additional hypotheses that explain the lower mortality rates associated with the higher serum uric acid levels. A low serum level of UA may be an indicator of malnutrition in the patients [9]. Similar to pre-dialysis BUN [22], lipid profiles are regarded as indicators of nutritional status in patients with dialysis, and low UA levels and pre-dialytic BUN levels are often surrogates of inadequate protein intake [4]. PEW, referring to uremic malnutrition, is caused by inadequate nutrient intake, nutrient loss during dialysis, and hyper-catabolism associated with dialysis [23]. Uric acid is an end product of protein metabolism, so PEW leads to low BMI hypocholesterolemia, a low level of pre-dialytic BUN, and even a low level of uric acid [4]. The nPCR is another well-recognized nutrition parameter. In our study, serum UA levels were significantly correlated with both pre-dialysis BUN (*p* < 0.001, Table 1) and the nPCR (*r* = 0.621, *p* < 0.001, Table 2). These findings suggest that serum UA is an alternative marker of protein intake. Therefore, it is possible that lower levels of serum uric acid resulted in a reduced total antioxidant capacity in our HD patients with CCI ≥ 4.

Clinically, serum biochemical parameters are used to assess health and pathological development. Biochemical indexes, somehow, are personally conditioned. Serum UA levels could be an example reflected in personalized medicine. There are certain risks in using medication to treat gout; generally speaking, the decision to treat it is based on the condition of the disease. For dialysis patients, when serum UA levels serve as a nutritional index instead of an inflammatory indicator in a population with multiple comorbidities, special treatment may not be required. To mitigate risk, urate-lowering agent administration could be the right treatment for this population. Alternatively, from the perspective of personalized medicine, the serum UA levels corresponding to the CCI can provide a reference for uric acid treatment in hemodialysis patients.

In the future, we need further studies to clarify the above paradoxical issues in HD patients with CCI < 4 and ≥4. Future treatments for gout may involve urate transport modulators or gene therapy. Except for hyperuricemia with CCI ≥ 4 as the nutritional index, we need to search for more biomarkers, including even those using artificial intelligence (AI)-based tools, to predict gout flare risk or treatment response. If possible, the integration of omics data (genomics, proteomics, and metabolomics) into dialysis and gout management would be an ideal approach.

This study included HD patients and measured the mean dialysis dose between months 4 and 9 of HD. This may have prevented survival bias and ensured stable measurement of the dialysis dose. This study has its limitations. First, this is an observational study, and a causal relationship between serum UA, the CCI, and clinical outcomes cannot be established. Second, the idea that uric acid reflects nutrition and inflammation is feasible but weak once no direct nutritional markers beyond albumin/PCR were presented (BMI, PEW score, prealbumin). Third, the key variables, such as residual urine, blood pressure, vascular-access type, time-varying Kt/V, phosphate-binder use, and urate-lowering therapy are not in the models. Lastly, clinical relevance is important because inflammation and malnutrition have pathological impacts, but we did not prove inflammation and malnutrition in any way.

## 5. Conclusions

In summary, low UA levels of serum uric acid in our HD patients were associated with worse mortality with CCI ≥ 4; paradoxically, high levels were associated with worse mortality with CCI < 4. High UA was not a risk in those with CCI ≥ 4, but low UA should be prevented in all HD patients. A personalized approach using CCI and corresponding serum UA levels offers a key reference for managing UA in hemodialysis patients.

## Figures and Tables

**Table 1:** Demographic data of incident hemodialysis patients in the cohort.

		UA (Pre-Dialysis)						
Variables	All	<5.8	5.8–6.5	6.5–7.1	7.1–7.7	7.7–8.6	>8.6	*p* Value
Demographics								*-*
No. of patients	2615	428 (16.4%)	448 (17.1%)	446 (17.1%)	436 (16.7%)	425 (16.3%)	432 (16.5%)	
Age (years)	59.1 (14.2)	64.9 (14.1)	61.7 (14.0)	59.4 (13.9)	57.7 (13.9)	56.5 (13.5)	54.0 (13.1)	<0.001 *
Gender (female %)	1317 (50.4%)	247 (57.7%)	267 (59.6%)	220 (49.3%)	197 (45.2%)	191 (44.9%)	195 (45.1%)	<0.001 *
Hepatitis	361 (13.8%)	58 (13.6%)	58 (12.9%)	70 (15.7%)	63 (14.4%)	58 (13.6%)	54 (12.5%)	0.753
CHF	850 (32.5%)	121 (28.3%)	158 (35.3%)	151 (33.9%)	115 (26.4%)	149 (35.1%)	156 (36.1%)	0.107
IHD	439 (16.8%)	61 (14.3%)	63 (14.1%)	86 (19.3%)	67 (15.4%)	86 (20.2%)	76 (17.6%)	0.038 *
Stroke	194 (7.4%)	32 (7.5%)	37 (8.3%)	40 (9.0%)	28 (6.4%)	30 (7.1%)	27 (6.3%)	0.239
Cancer	161 (6.2%)	25 (5.8%)	34 (7.6%)	32 (7.2%)	25 (5.7%)	23 (5.4%)	22 (5.1%)	0.219
DM	1261 (48.2%)	206 (48.1%)	232 (51.8%)	238 (53.4%)	207 (47.5%)	206 (48.5%)	172 (39.8%)	0.004 *
Hypertension	1831 (70.0%)	261 (61.0%)	315 (70.3%)	327 (73.3%)	305 (70.0%)	314 (73.9%)	309 (71.5%)	0.001 *
CCI	3.9 (1.7)	3.9 (1.7)	4.0 (1.8)	4.1 (1.8)	3.8 (1.6)	4.0 (1.7)	3.7 (1.5)	<0.001 *
Laboratory data								
WBCs (×1000/uL)	7.0 (2.3)	7.1 (2.5)	7.0 (2.4)	7.0 (2.3)	7.0 (2.1)	6.9 (2.1)	7.0 (2.3)	0.871
Hb (g/dL)	9.9 (1.2)	9.6 (1.1)	9.9 (1.2)	10.0 (1.2)	10.1 (1.2)	10.0 (1.3)	9.6 (1.2)	<0.001 *
Albumin (g/dL)	3.7 (0.4)	3.5 (0.5)	3.7 (0.4)	3.8 (0.4)	3.8 (0.4)	3.8 (0.3)	3.8 (0.4)	<0.001 *
Cholesterol (mg/dL)	187.0 (45.1)	174.7 (44.1)	183.0 (42.3)	184.4 (45.5)	190.1 (44.2)	192.1 (43.7)	198.1 (47.2)	<0.001 *
Glucose [AC] (mg/dL)	136.4 (60.8)	139.7 (64.8)	137.6 (59.2)	138.4 (60.0)	134.3 (57.5)	136.0 (59.0)	132.5 (63.9)	0514
Creatinine (mg/dL)	9.2 (2.8)	7.2 (2.4)	8.2 (2.4)	9.2 (2.5)	9.7 (2.5)	10.1 (2.7)	11.2 (2.7)	<0.001 *
K (mEq/L)	4.7 (0.7)	4.5 (0.7)	4.5 (0.7)	4.6 (0.7)	4.7 (0.6)	4.8 (0.6)	4.8 (0.7)	<0.001 *
Ca (mg/dL)	9.3 (0.8)	9.3 (0.9)	9.3 (0.7)	9.3 (0.7)	9.3 (0.8)	9.3 (0.8)	9.4 (0.9)	0.486
P (mg/dL)	5.0 (1.2)	4.2 (1.2)	4.7 (1.1)	4.9 (1.1)	5.2 (1.1)	5.2 (1.2)	5.7 (1.2)	<0.001 *
BW post-dialysis (kg)	56.7 (11.7)	51.7 (10.3)	54.2 (10.5)	56.3 (10.4)	57.2 (11.8)	59.6 (11.8)	61.2 (12.6)	<0.001 *
UF/BW ratio (%)	3.8 (1.5)	3.6 (1.5)	3.7 (1.5)	3.9 (1.6)	3.9 (1.4)	3.9 (1.5)	3.9 (1.5)	0.0030 *
BUN pre-HD (mg/dL)	70.3 (18.1)	59.2 (17.1)	65.4 (17.0)	68.0 (15.4)	72.2 (15.8)	74.8 (17.0)	82.5 (17.3)	<0.001 *
URR	0.7 (0.1)	0.7 (0.1)	0.7 (0.1)	0.7 (0.1)	0.7 (0.1)	0.7 (0.1)	0.7 (0.1)	<0.001 *
Kt/V (Gotch)	1.3 (0.2)	1.3 (0.2)	1.3 (0.2)	1.3 (0.2)	1.3 (0.2)	1.3 (0.2)	1.2 (0.2)	<0.001 *
nPCR	1.2 (0.3)	1.1 (0.3)	1.1 (0.3)	1.1 (0.3)	1.2 (0.3)	1.2 (0.3)	1.2 (0.3)	<0.001 *
CTR (%)	50.3 (6.5)	51.5 (6.7)	50.8 (6.5)	50.2 (6.3)	49.7 (6.6)	49.4 (6.5)	50.2 (6.2)	<0.001 *
Outcomes								
All-cause mortality	1115 (42.6%)	247 (57.7%)	198 (44.2%)	198 (44.4%)	162 (37.2%)	165 (38.8%)	145 (33.6%)	<0.001 *

Abbreviations. CHF, congestive heart failure; IHD, ischemic heart disease; DM, diabetes mellitus; CCI, Charlson comorbidity index; WBCs, white blood cells; Hb, hemoglobin; K, potassium; Ca, calcium; P, phosphate; BW, body weight; UF, ultrafiltration; BUN, blood urea nitrogen; HD, hemodialysis; URR, urea reduction ratio; nPCR, normalized protein catabolic rate; CTR, cardiac/thoracic ratio. Data are presented as mean (standard error), median (interquartile range), or count (percentage). * (*p* < 0.05) indicates a significant difference.

**Table 2:** Multivariate linear regression (fully adjusted model for UA level) (continuous).

Variables	β Coefficient	95% CI β Coefficient	*p*
Gender (female vs. male)	−0.164	−0.283 to −0.045	0.007
Age at dialysis (year)	−0.008	−0.012 to −0.004	<0.001
Entry year (late vs. early)	−0.211	−0.324 to −0.099	<0.001
Hepatitis	−0.070	−0.215 to 0.075	0.347
CHF	0.032	−0.079 to 0.144	0.568
Cancer	0.092	−0.115 to 0.298	0.385
DM	−0.188	−0.307 to −0.070	0.002
Hypertension	0.051	−0.062 to 0.164	0.377
Post-dialytic body weight (kg)	0.012	0.007 to 0.017	<0.001
Kt/V (Gotch)	−0.524	−0.804 to −0.243	<0.001
UF/BW ratio100	0.026	−0.009 to 0.061	0.147
nPCR	0.612	0.420 to 0.804	<0.001
W.B.C. (1000/uL)	0.016	−0.007 to 0.040	0.176
Hemoglobin (g/dL)	−0.007	−0.052 to 0.038	0.745
Albumin (g/dL)	0.256	0.102 to 0.409	0.001
Cholesterol log	1.361	0.839 to 1.883	<0.001
Glucose [AC] (mg/dL)	0.000	−0.001 to 0.001	0.761
P (mg/dL)	0.267	0.220 to 0.314	<0.001
Total Ca (mg/dL)	−0.008	−0.074 to 0.058	0.814
PTH hormone log	0.123	0.044 to 0.202	0.002

UA: uric acid, PTH: parathyroid, other abbreviations are the same as in Table 1. Data are presented as in Table 1.

**Table 3:** HR of UA sextiles for total mortality, Charlson ≥ 4 and Charlson < 4 in 2 groups.

UA						
Sextile	1	2	3	4	5	6
Variables	<5.8	5.8–6.5	6.5–7.1	7.1–7.7	7.7–8.6	>8.6
Number	428	448	446	436	425	432
Total (*n* = 2615)						
unadjusted	2.22 (1.83–2.69) **	1.57 (1.29–1.92) **	1.48 (1.21–1.81) **	1.20 (0.98–1.48)	1.25 (1.02–1.54) *	1 (reference)
fully adjusted	1.31 (1.06–1.63) *	1.09 (0.88–1.36)	1.21 (0.98–1.49)	1.07 (0.86–1.32)	1.20 (0.97–1.48)	1 (reference)
Charlson ≥ 4 (*n* = 1507)						
unadjusted	2.67 (2.15–3.32) **	1.72 (1.38–2.15) **	1.64 (1.32–2.06) **	1.39 (1.11–1.76) *	1.38 (1.10–1.74) *	1 (reference)
fully adjusted	1.53 (1.20–1.95) **	1.19 (0.93–1.51)	1.37 (1.08–1.72) *	1.22 (0.96–1.55)	1.34 (1.06–1.69) *	1 (reference)
Charlson < 4 (*n* = 1108)						
unadjusted	1.88 (1.20–2.94) *	1.54 (0.97–2.45)	1.27 (0.79–2.06)	1 (reference)	0.96 (0.57–1.63)	1.27 (0.79–2.04)
fully adjusted	1.21 (0.75–1.95)	1.51 (0.94–2.43)	1.35 (0.82–2.23)	1 (reference)	1.18 (0.69–2.04)	1.61 (1.01–2.38) *

HR: hazard ratio, *: <0.05, **: <0.01, other abbreviations are the same as in Table 2. Data are presented as in Table 2.

## Data Availability

Data are contained within the article and Supplementary Materials.

## Supplementary Materials

The following supporting information can be downloaded at https://www.mdpi.com/article/10.3390/jpm15070305/s1: Table S1: Charlson comorbidity index (CCI); Table S2: Demographic data of incident hemodialysis patients in UA quintile group; Table S3: HR of UA quintiles for total mortality, Charlson ≥ 4 and Charlson < 4 in 2 groups. Figure S1A. Through modeling UA as a continuous variable with restricted cubic splines and plotting it using CCI strata, it revealed the lowest HR in all patients at a UA of 9.3; Figure S1B: As the same model of Figure S1A, in patients with CCI < 4, the lowest HR was at a UA of 7.9; Figure S1C: As the same model of Figure S1A, in patients with CCI ≥ 4, the lowest HR was at a UA of 9.4.

## Abbreviations

The following abbreviations are used in this manuscript:

UA	Uric acid
ESKD	End-stage kidney disease
CCI	Charlson Comorbidity Index
HR	Hazard ratio
CI	Confidence interval
PEW	Protein-energy wasting
CKD	Chronic kidney disease
HD	Hemodialysis
WBC	White blood cell
Hb	Hemoglobin
HbA1c	Hemoglobin A1c
LDL	Low-density lipoprotein cholesterol
AMP	Adenosine monophosphate
MIA	Malnutrition–inflammation–atherosclerosis
MS	Metabolic syndrome
DM	Diabetes mellitus
BW	Body weight
UF	Ultrafiltration
BUN	Blood urea nitrogen
nPCR	Normalized protein catabolic rate
AC	Ante cibum
K	Potassium
Ca	Calcium
P	Phosphate
URR	Urea reduction ratio
CTR	Cardiac/thoracic ratio
PTH	Parathyroid
eGFR	Estimated glomerular filtration rate
NHI	National Health Insurance
OR	Odds ratio
RAAS	Renin–angiotensin–aldosterone system
ROS	Oxygen free radicals
BSA	Body surface area
KDIGO	Kidney Disease: Improving Global Outcomes
CHF	Congestive heart failure
BCG	Bromocresol green
KDOQI	Kidney Disease Outcomes Quality Initiative
SD	Standard deviation
SPSS	Statistical Product and Service Solutions
