# Supplementary material for: Serum Uric Acid Level as an Estimated Parameter That Predicts All-Cause Mortality in Patients with Hemodialysis
Source: J Pers Med. 2025 Jul 11;15(7):305. doi: 10.3390/jpm15070305 (PMC12298383; doi:10.3390/jpm15070305)
Supplement: Supplementary file 1 [file jpm-15-00305-s001.zip › jpm-3595500-supplementary.pdf]

Table S1. Charlson comorbidity index (CCI).

Conditions	Assigned weights for each condition
Myocardial infarction	1
Congestive heart failure	1
Peripheral vascular disease	1
Cerebrovascular disease	1
Dementia	1
Chronic pulmonary disease	1
Connective tissue disease	1
Ulcer disease	1
Mild liver disease	1
Diabetes mellitus	1
Hemiplegia	2
Moderate or severe renal disease (including ESRD)	2
Diabetes with end organ damage	2
Any tumor without metastasis	2
Leukemia	2
Lymphoma	2
Moderate or severe liver disease	3
Metastatic solid tumor	6
Acquired inmmunodeficiency syndrome	6

ESRD, end stage renal disease.

Charlson comorbidity index	Estimated 10-year survival
0	98%
1	96%
2	90%
3	77%
4	53%
5	21%
6	2%
≥7	0

Table S2. Demographic data of incident hemodialysis patients in UA quintiles group.

**Variables**	**UA**						
	All	<6	6-7	7-8	8-9	>9	*p* value
No. of patients	2615	558 (21.3%)	742 (28.4%)	643 (24.6%)	419 (16.0%)	253 (9.7%)	-
**Demographics**							
Age (years)	59.1 (14.2)	64.8 (13.7)	60.0 (14.1)	58.0 (13.8)	55.3 (13.2)	52.5 (13.0)	<0.001
Gender, (female %)	1317 (50.4%)	326 (58.4%)	395 (53.2%)	287 (44.6%)	190 (45.3%)	119 (47.0%)	<0.001
Hepatitis	361 (13.8%)	76 (13.6%)	106 (14.3%)	94 (14.6%)	56 (13.4%)	29 (11.5%)	0.7811
CHF	850 (32.5%)	185 (33.2%)	245 (33.0%)	194 (30.2%)	138 (32.9%)	88 (34.8%)	0.6578
IHD	439 (16.8%)	80 (14.3%)	130 (17.5%)	111 (17.3%)	77 (18.4%)	41 (16.2%)	0.4618
Stroke	194 (7.4%)	49 (8.8%)	60 (8.1%)	42 (6.5%)	29 (6.9%)	14 (5.5%)	0.3814
Cancer	161 (6.2%)	40 (7.2%)	51 (6.9%)	36 (5.6%)	26 (6.2%)	8 (3.2%)	0.2050
DM	1261 (48.2%)	303 (54.3%)	373 (50.3%)	310 (48.2%)	186 (44.4%)	89 (35.2%)	<0.001
Hypertension	1831 (70.0%)	359 (64.3%)	531 (71.6%)	459 (71.4%)	304 (72.6%)	178 (70.4%)	0.0235

CCI	3.9 (1.7)	4.1 (1.7)	4.0 (1.8)	3.9 (1.7)	3.8 (1.5)	3.6 (1.6)	<0.001
**Laboratory data**							
WBC (x1000/ul)	7.0 (2.3)	7.1 (2.5)	7.0 (2.3)	7.0 (2.1)	6.9 (2.1)	6.9 (2.3)	0.514
Hb (g/dl)	9.9 (1.2)	9.6 (1.1)	9.9 (1.2)	10.1 (1.3)	9.8 (1.2)	9.6 (1.1)	<0.001
Albumin (g/dl)	3.7 (0.4)	3.5 (0.5)	3.8 (0.4)	3.8 (0.4)	3.8 (0.4)	3.9 (0.3)	<0.001
Cholesterol (mg/dl)	187.0 (45.1)	176.4 (44.0)	184.1 (44.2)	190.2 (42.7)	192.2 (47.6)	202.5 (45.7)	<0.001
Glucose[AC] (mg/dl)	136.4 (60.8)	143.7 (65.6)	135.6 (58.1)	135.5 (57.7)	132.1 (61.0)	132.0 (63.6)	0.019
Creatinine (mg/dl)	9.2 (2.8)	7.3 (2.5)	8.8 (2.4)	9.7 (2.5)	10.6 (2.6)	11.6 (2.7)	<0.001
K (mEq/l)	4.7 (0.7)	4.5 (0.7)	4.6 (0.7)	4.7 (0.6)	4.8 (0.6)	4.9 (0.7)	<0.001
Ca (mg/dl)	9.3 (0.8)	9.3 (0.9)	9.3 (0.7)	9.3 (0.7)	9.3 (0.8)	9.3 (0.9)	0.789
P (mg/dl)	5.0 (1.2)	4.3 (1.2)	4.8 (1.1)	5.2 (1.1)	5.4 (1.2)	5.8 (1.2)	<0.001
BW post dialysis (kg)	56.7 (11.7)	52.1 (10.2)	55.6 (10.6)	57.9 (11.6)	60.0 (12.7)	61.3 (12.0)	<0.001
UF/BW ratio (%)	3.82 (1.50)	3.67 (1.48)	3.82 (1.59)	3.84 (1.41)	3.89 (1.50)	4.03 (1.47)	0.0210
BUN pre-HD (mg/dl)	70.3 (18.1)	60.7 (18.3)	66.6 (15.3)	72.3 (16.0)	77.8 (16.5)	84.9 (18.0)	<0.001
URR	0.7 (0.1)	0.7 (0.1)	0.7 (0.1)	0.7 (0.1)	0.7 (0.1)	0.7 (0.1)	<0.001
Kt/V (Gotch)	1.3 (0.2)	1.3 (0.2)	1.3 (0.2)	1.3 (0.2)	1.3 (0.2)	1.2 (0.2)	<0.001
nPCR	1.2 (0.3)	1.1 (0.3)	1.1 (0.3)	1.2 (0.3)	1.2 (0.3)	1.2 (0.3)	<0.001
CTR (%)	50.3 (6.5)	51.52 (6.58)	50.30 (6.40)	49.79 (6.61)	49.81 (6.35)	49.85 (6.29)	<0.001
**Outcomes**							
All-cause mortality	1115 (42.6%)	315 (56.5%)	323 (43.5%)	241 (37.5%)	156 (37.2%)	80 (31.6%)	<0.001

Abbreviations. CHF, congestive heart failure; IHD, ischemic heart disease; DM, Diabetes mellitus; CCI, Charlson comorbidity index; WBC, white blood cells; Hb, hemoglobin; K, potassium; Ca, calcium; P, phosphate; BW, body weight; UF, ultrafiltration; BUN, blood urea nitrogen; HD, hemodialysis; URR, urea reduction ratio; nPCR, normalized protein catabolic rate; CTR, Cardiac/thoracic ratio. Data are presented as mean (standard error), median (interquartile range), or count (percentage%). * (P<0.05) indicates a significant difference.

Table S3. HR of UA quintiles for total mortality, Charson ≥ 4 and Charson < 4 by 2 groups.

**Variables**	**UA**				
	<6	6-7	7-8	8-9	>9
Number					
Total (n=2615)					
unadjusted	2.25 (1.80-2.82)**	1.55 (1.24-1.94)**	1.28 (1.02-1.61)*	1.19 (0.93-1.52)	1 (reference)
fully-adjusted	1.10 (0.86-1.42)	1.17 (0.92-1.48)	1.03 (0.81-1.30)	1.07 (0.83-1.37)	1 (reference)
charlson ≥4 (n=1507)					
unadjusted	2.49 (1.92-3.24)**	1.78 (1.37-2.31)**	1.48 (1.14-1.94)*	1.31 (0.99-1.74)	1 (reference)
fully-adjusted	1.32 (0.99-1.76)	1.39 (1.06-1.82)*	1.23 (0.94-1.62)	1.22 (0.91-1.62)	1 (reference)
charlson <4 (n=1108)					
unadjusted	1.83 (1.22-2.75)*	1.36 (0.93-1.98)	1 (reference)	1.03 (0.64-1.64)	1.33 (0.83-2.15)
fully-adjusted	1.15 (0.74-1.79)	1.40 (0.95-2.07)	1 (reference)	1.26 (0.78-2.03)	1.99 (1.18-3.36)*

HR: hazard ratio, *:<0.05, **:<0.01, ***:<0.001, other abbreviations are the same as in Table S2. Data are presented as in Table S2.

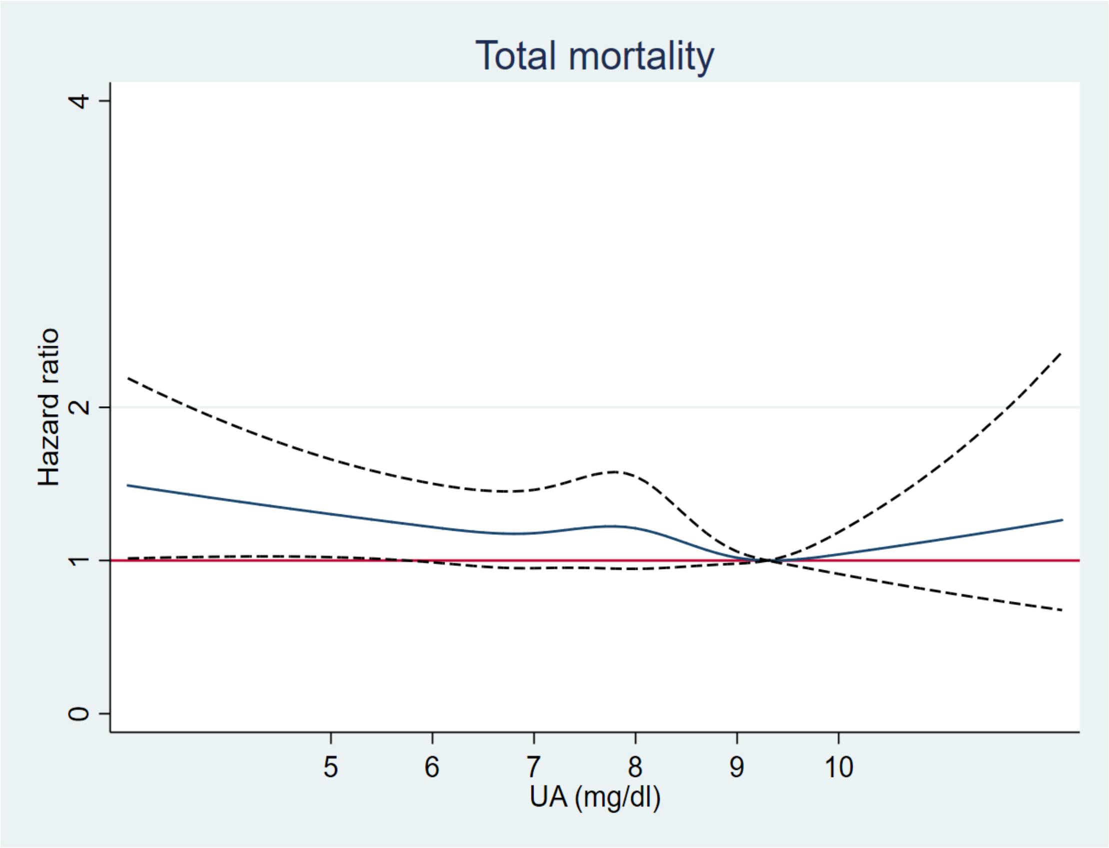

Supplement Figure S1A. Through modeling UA as a continuous variable with restricted cubic splines and plotting it using CCI strata, it revealed the lowest HR in all patients at a UA of 9.3.

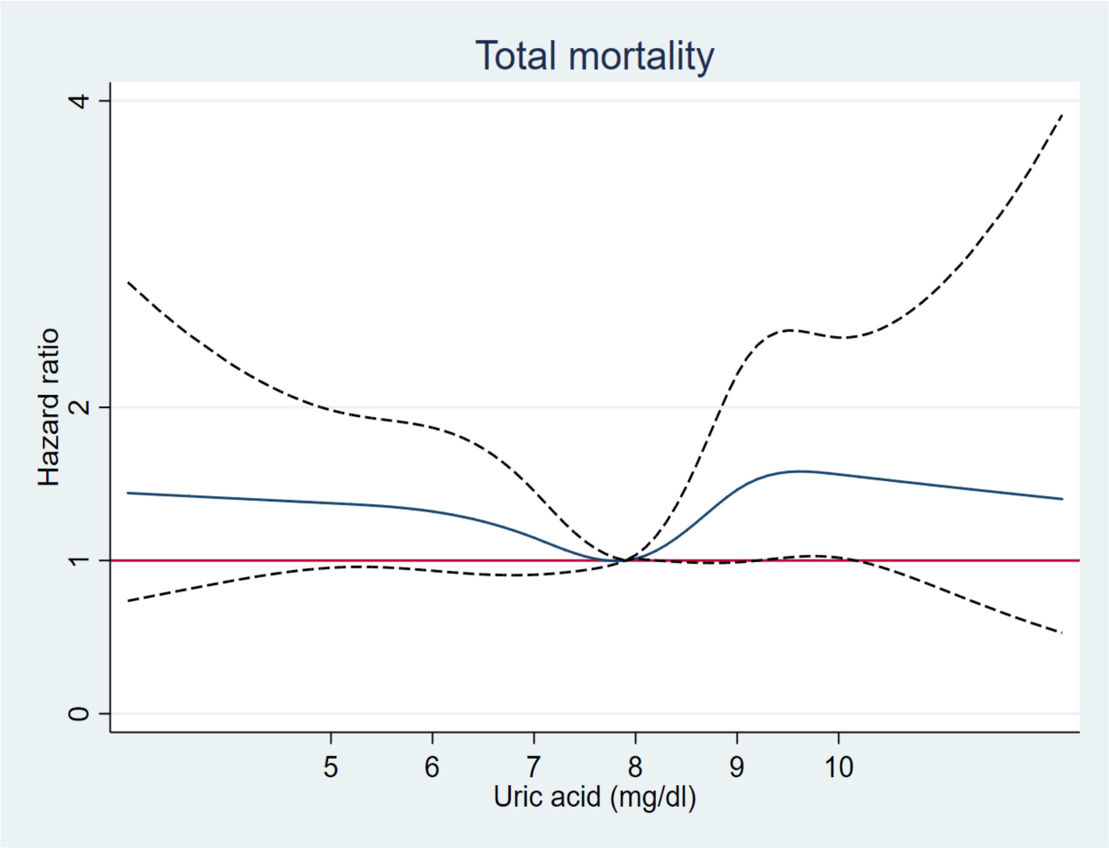

Supplement Figure S1B. As the same model of figure S1A, in patients with CCI < 4, the lowest HR was at a UA of 7.9.

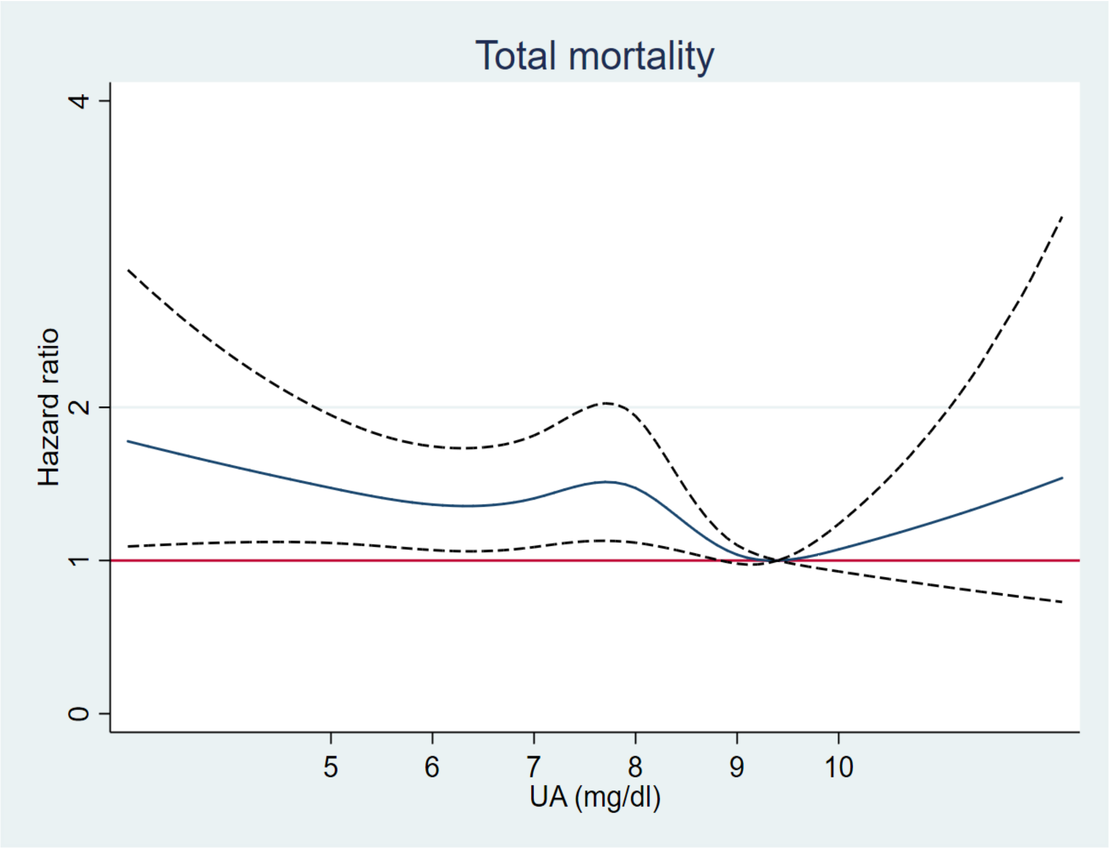

Supplement Figure S1C. As the same model of figure S1A, in patients with CCI ≥ 4, the lowest HR was at a UA of 9.4.
